# Supplementary material for: Network‐based visualisation reveals new insights into transposable element diversity
Source: Mol Syst Biol. 2021 Jun 25;17(6):e9600. doi: 10.15252/msb.20209600 (PMC8226279; doi:10.15252/msb.20209600)
Supplement: Supplementary file 1 — Appendix [file MSB-17-e9600-s002.pdf]

Appendix

Contents

1 Figure S1 3

2 Figure S2 4

3 Figure S3 5

4 Figure S4 6

5 Figure S5 7

6 Figure S6 8

7 Figure S7 9

8 Figure S8 10

9 Figure S9 11

10 Figure S10 12

11 Figure S11 13

12 Figure S12 14

1 Figure S1

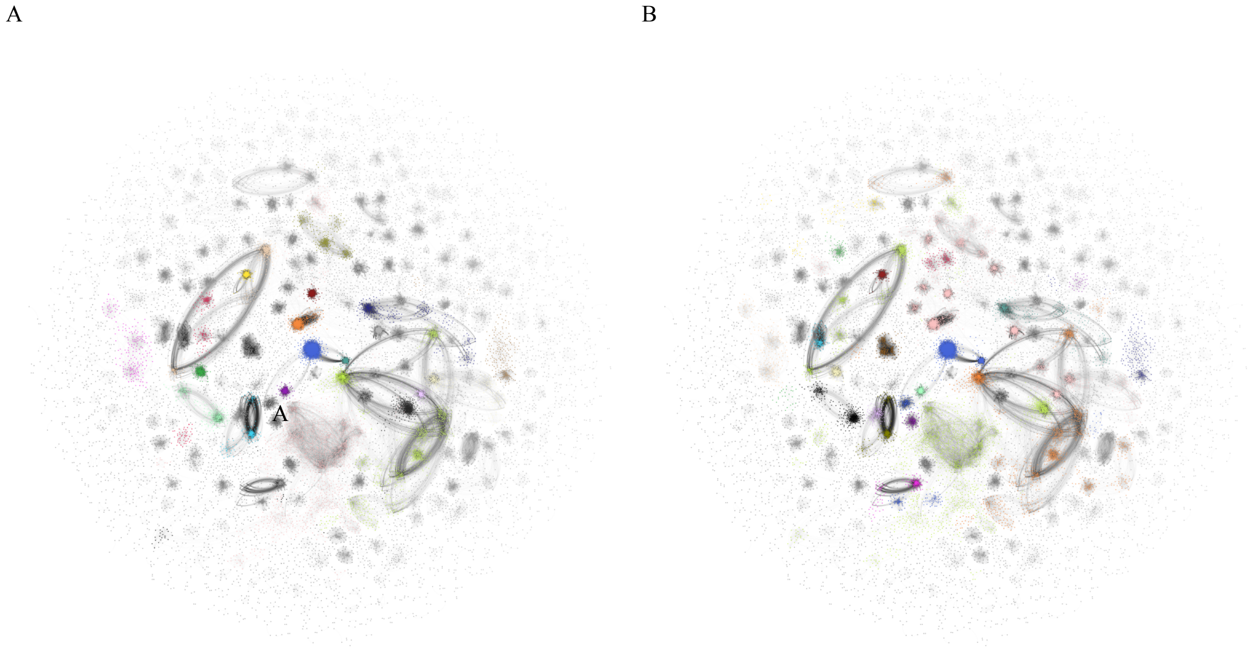

Figure S1: Comparison between different clustering algorithms.

A Network visualization of the sequence similarity network using the openOrd layout algorithm and colouring nodes by their modularity class. Smaller clusters are coloured in grey.
B Network visualization of the sequence similarity network using the openOrd layout algorithm and colouring nodes by their connected component. Smaller clusters are coloured in grey.

2 Figure S2

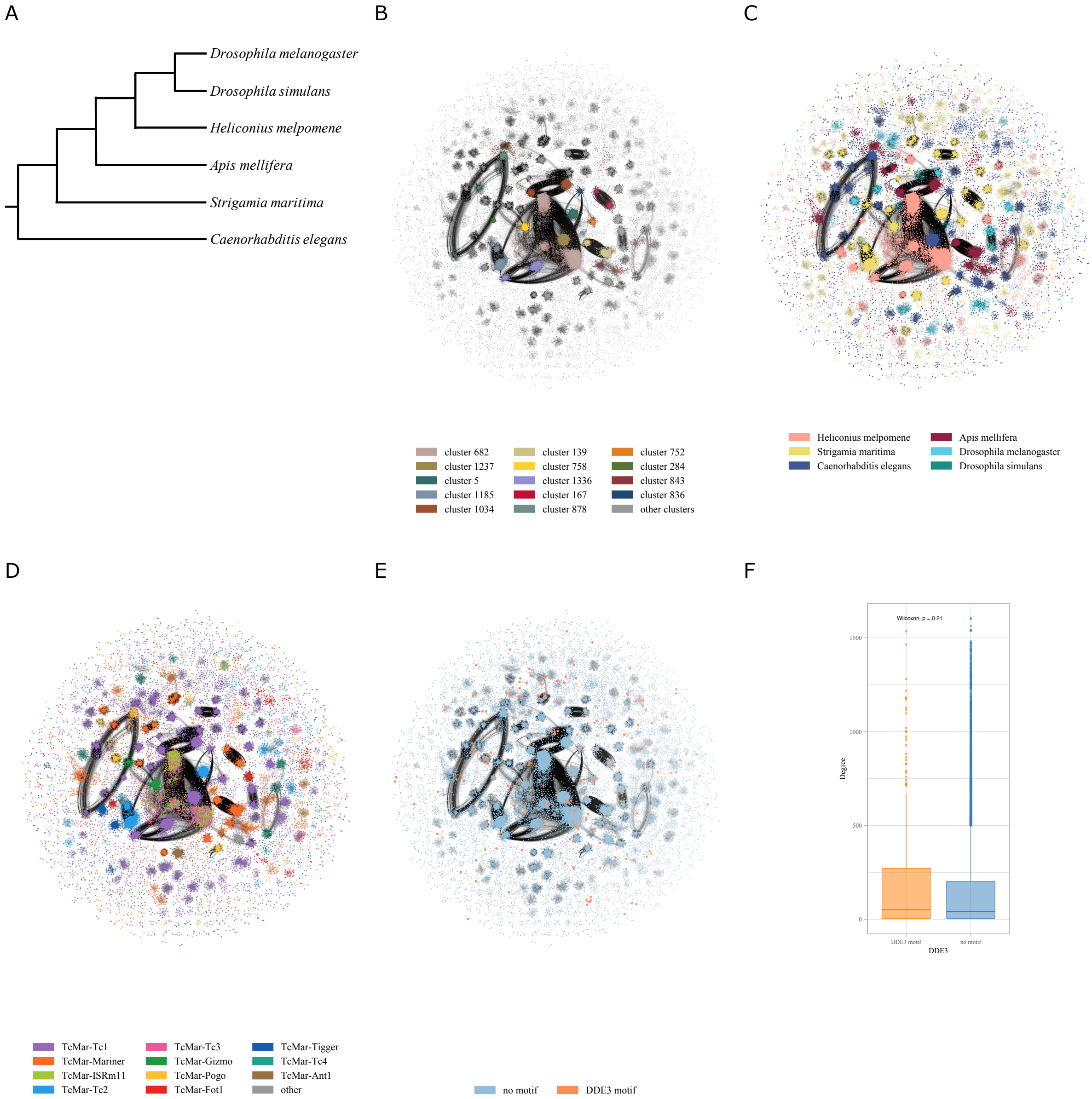

Figure S2: TcMar/mariner Sequence Similarity Network of Insect species.

A A phylogenetic tree showing the evolutionary relationship between the six species in the Insect SSN.
B Visualization of the network using the openOrd layout algorithm. The SSN consists of the five insects *Drosophila melanogaster*, *Drosophila simulans*, *Heliconius melpomene*, *Apis mellifera*, and *Strigamia maritima* and the nematode *C. elegans*. Each node in the network represents a TE copy from the respective genome and the edge weights indicate sequence similarity. The nodes belonging to the 14 largest clusters are coloured by cluster identity and all other nodes are coloured in grey.
C The SSN with all nodes coloured according to the sequences' respective origin genome of origin.
D The SSN with nodes coloured according to the family of the TE.
E The sequences with a DDE3 transposase domain are highlighted (orange).
F Boxplot showing the difference in connectivity between 497 sequences containing a DDE3 motif and 28619 sequences without a motif. DDE3 containing sequences have a significantly higher connectivity (p=0.21, Wilcoxon). The box outlines the interquartile range (IQR) with the median shown as a line, whiskers indicate the fences (lower fence: lowest value at most Q1-1.5*IQR, upper fence: largest value no further than Q3+1.5*IQR) and data beyond the whiskers are drawn as individual points (outliers).

3 Figure S3

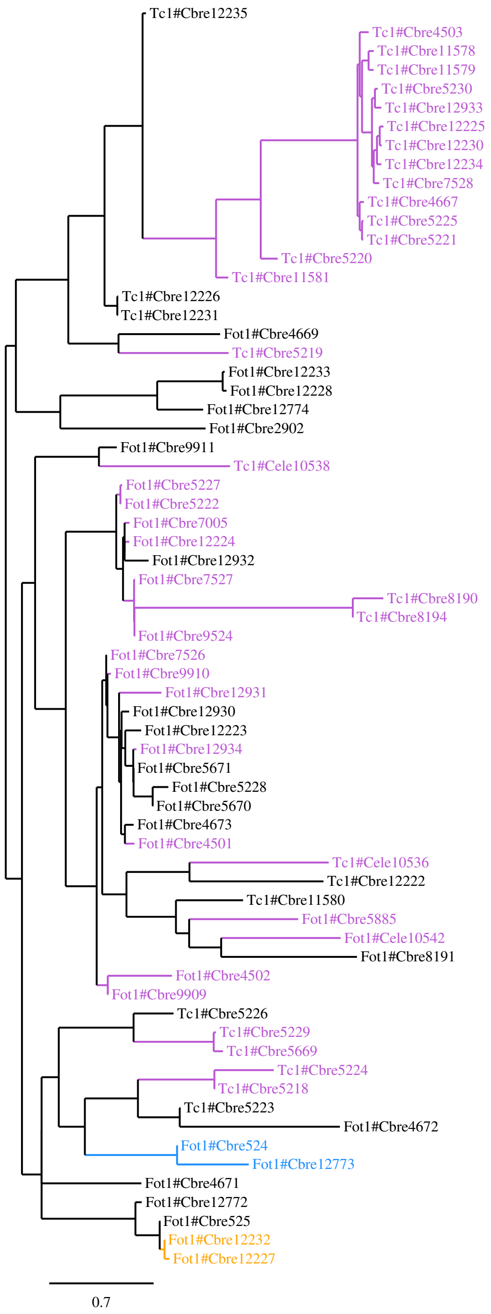

Figure S3: Multiple sequence alignment of the mixed cluster mc476.

Reconstructed phylogenetic tree of all sequences from the SSN that are classified as Fot1 or Tc1 TE subfamilies. Sequences with a C. elegans rbc-1 blast hits are highlighted in purple. Sequences that are not in the mixed cluster c476 are coloured differently (blue, orange).

4 Figure S4

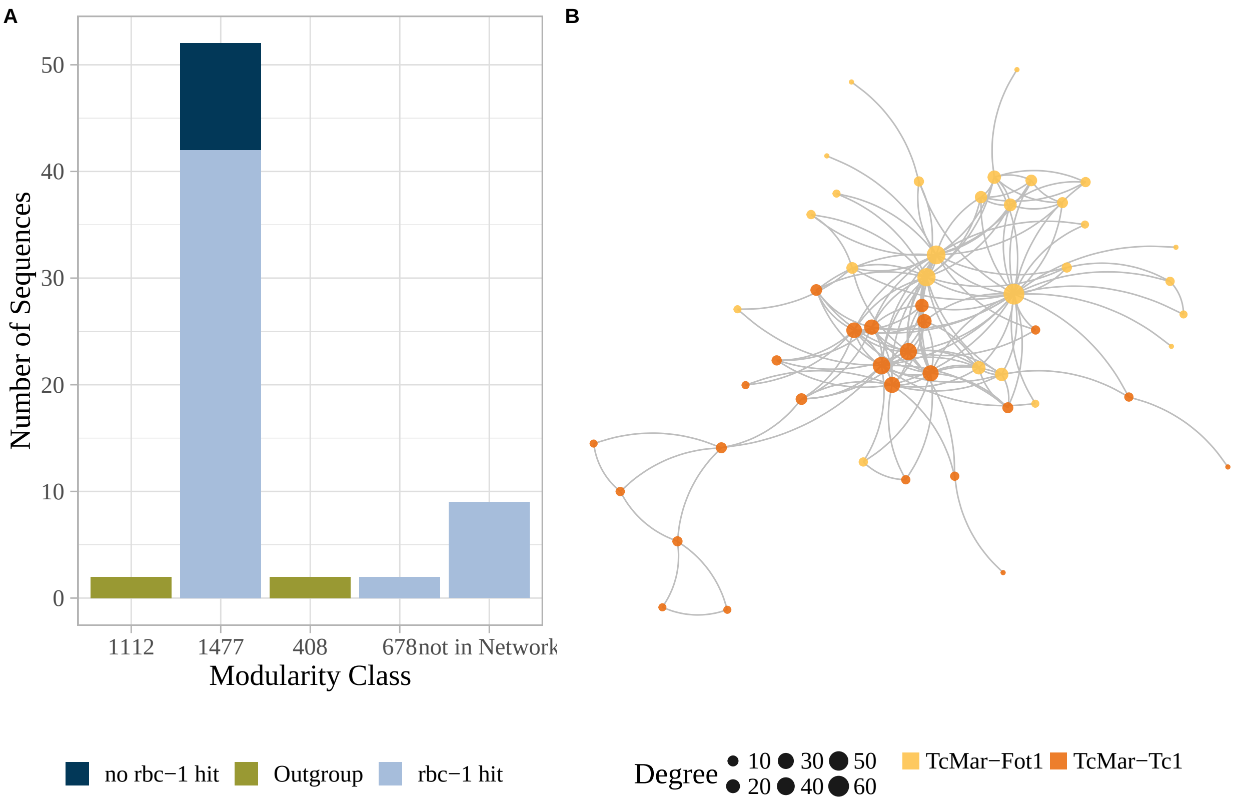

Figure S4: Network Visualization of the mixed cluster with masked *rbc-1* hits.

A Barplot showing the the new clusters for all sequences in mc476 as well as the four outgroup sequences. Sequences classified as ”not in network” did not have sufficient length after *rbc-1* masking.
B Network visualization of the sequence similarity between the sequences from the mixed cluster mc476 when all *rbc-1* hits are masked. The edges indicate sequence similarity, the node size is proportional to the node degree (number of connections) and the colour visualizes the TE family each sequence belongs to.

5 Figure S5

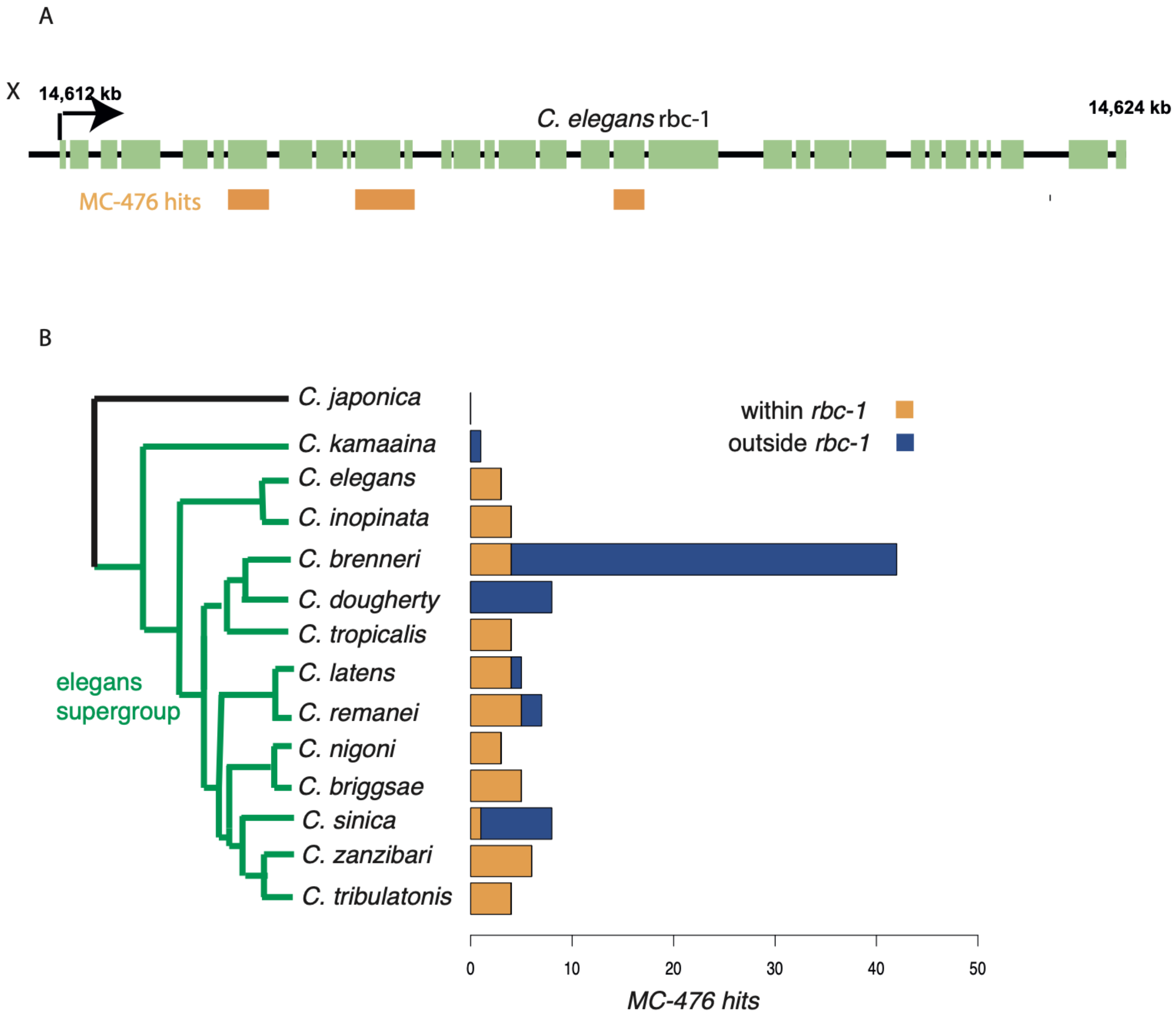

Figure S5: Evolution of *rbc-1* in *Caenorhabditis*.

A Genome browser snapshot showing the intron-exon structure and location of *C. elegans rbc-1*. The genomic positions of the *C. elegans* members of mc476 are illustrated.
B The number of TEs homologous to mc476 members across *Caenorhabditis* species, with the number of these that are within and outside of the *rbc-1* gene illustrated. A cladogram based on recent phylogenetic analysis is shown (Stevens et al, 2019).

6 Figure S6

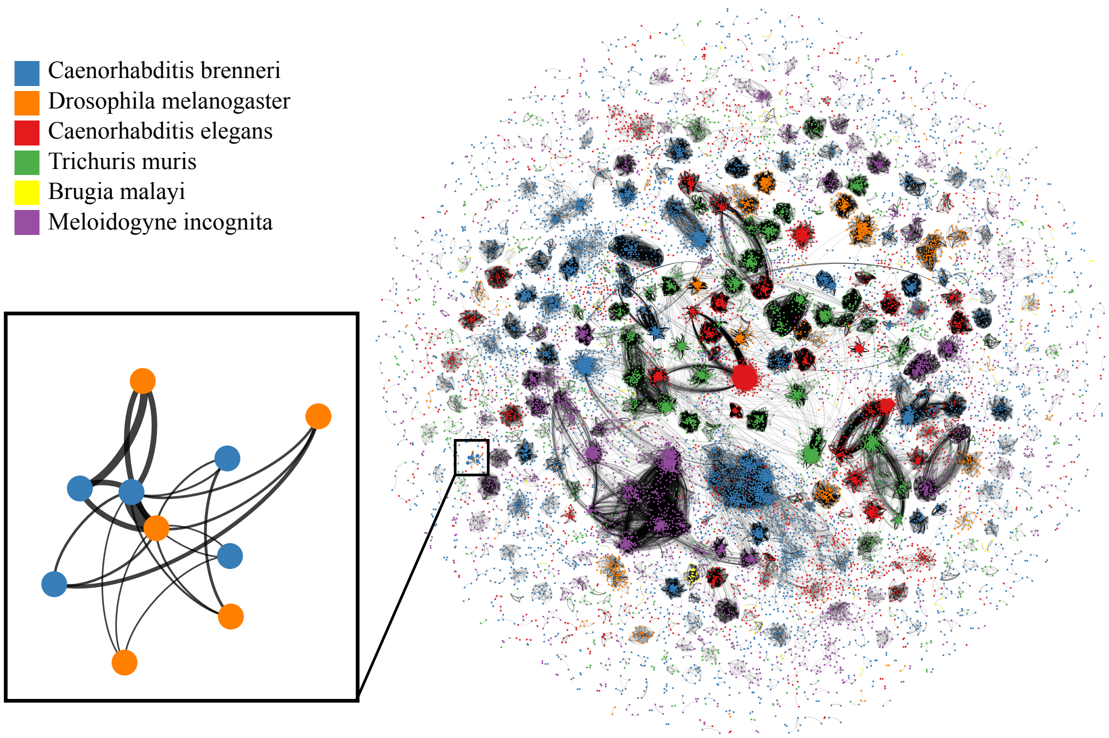

Figure S6: Artificial Horizontal TE transfer visualized in the TcMar/mariner SSN.

Network visualization of the TcMar/mariner SSN using the openOrd layout algorithm with five closely related TE sequences from *D. melanogaster* added to *C. brenneri* to simulate horizontal TE transfer. All nodes are coloured by species. The transferred TEs are emphasised by a larger node size. The cluster containing the original *D. melanogaster* TEs and the transferred *C. brenneri* TEs is highlighted with a black box in the network and a closeup is shown as well. The edge line thickness is proportional to blast bit score.

7 Figure S7

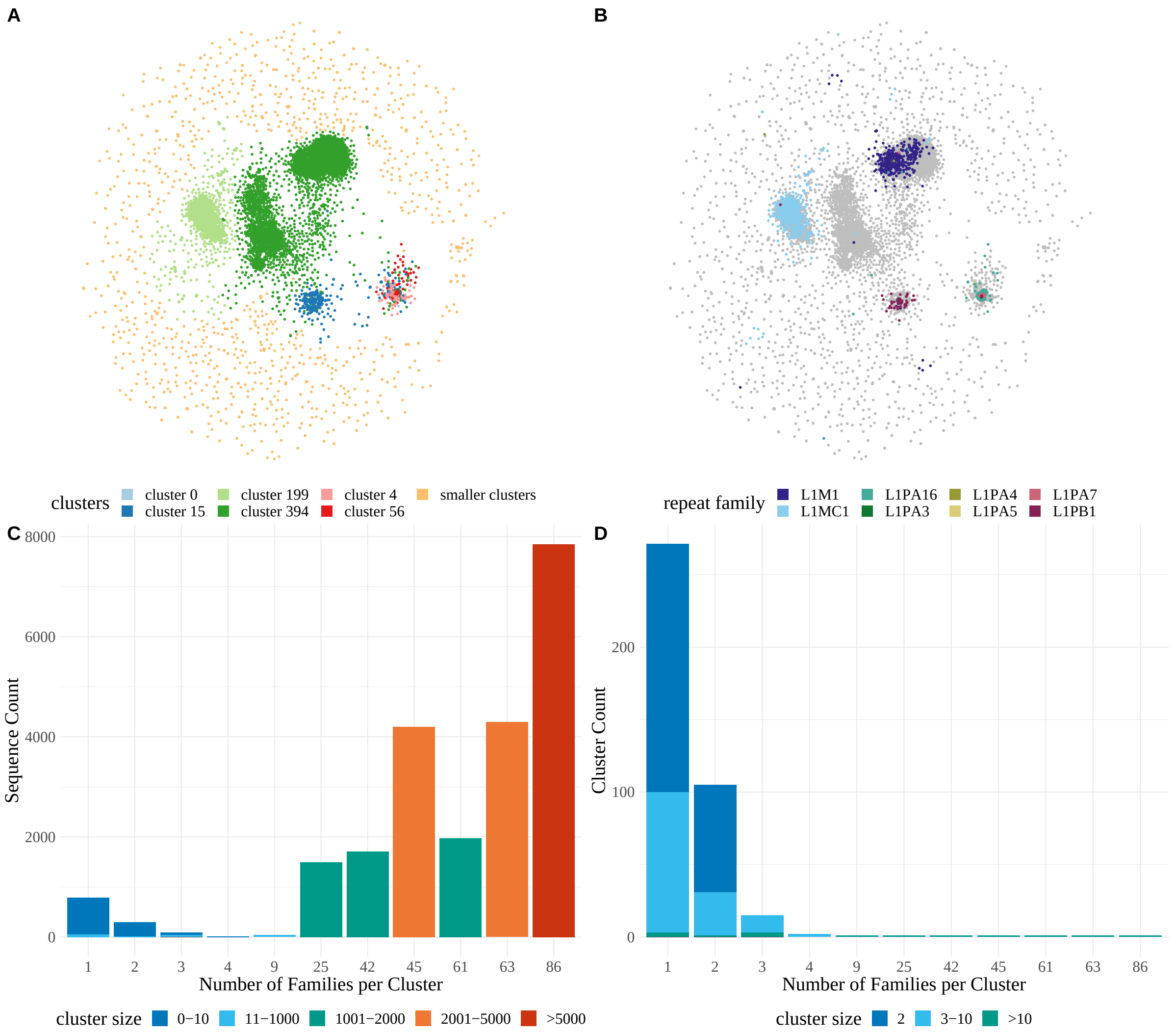

Figure S7: Sequence similarity network of human LINE-1 TEs.

A Visualisation of the human LINE-1 SSN using the openOrd layout algorithm. Each node in the network represents a TE copy. The nodes belonging to the six largest clusters are coloured by cluster identity. All other nodes are coloured yellow.
B Visualisation of the human LINE-1 SSN using the openOrd layout algorithm. Nodes belonging to the most common repeat families are coloured according to their repeat family classification, all other nodes are coloured in grey.
C Barplot depicting the number of sequences in clusters with different numbers of families. Clusters are coloured by cluster size.
D Barplot showing the number of clusters and number of families in each cluster. The clusters are coloured by cluster size.

8 Figure S8

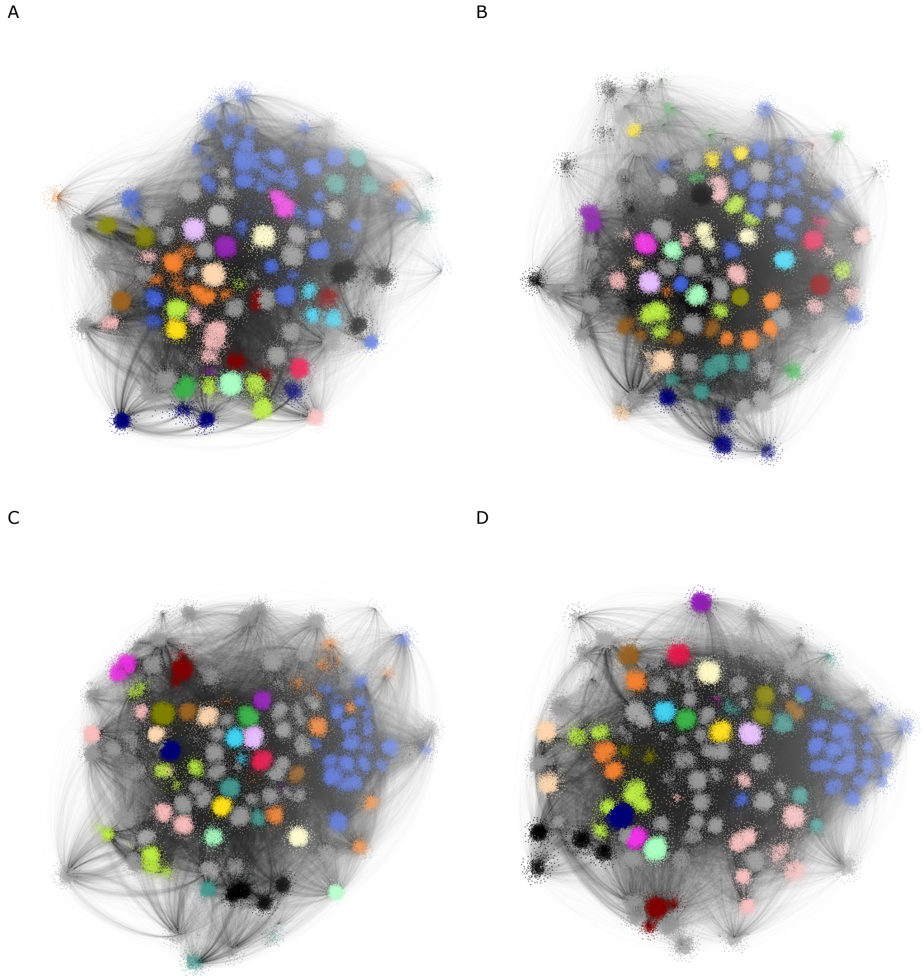

E

Adjusted Rand Index	TE count	TE length	% masked	% genome
TE count	1	0.74	0.58	0.62
TE length		1	0.76	0.80
% masked			1	0.91
% genome				1

Figure S8: Comparison of different TE content measurements.

A Network visualization of the TE count bipartite network using the openOrd layout algorithm. Genome nodes are connected to TE consensus sequences. The edge weight is proportional to TE count.
B Network visualization of the TE length bipartite network using the openOrd layout algorithm. Genome nodes are connected to TE consensus sequences. The edge weight is proportional to TE coverage in base pairs. The nodes are coloured according to their cluster. Smaller clusters are coloured grey. The nodes are coloured according to their cluster. Smaller clusters are coloured grey. This is the same representation as in Figure 3A, for ease of comparison.
C Network visualization of the % genome bipartite network using the openOrd layout algorithm. Genome nodes are connected to TE consensus sequences. The edge weight is proportional to TE coverage as a percentage of the genome. The nodes are coloured according to their cluster. Smaller clusters are coloured grey.
D Network visualization of the %masked bipartite network using the openOrd layout algorithm. Genome nodes are connected to TE consensus sequences. The edge weight indicates the coverage as a percentage of all TEs in the genome. The nodes are coloured according to their cluster. Smaller clusters are coloured grey.
E Table showing the calculated adjusted rand index between the bipartite networks with different TE content measures.

9 Figure S9

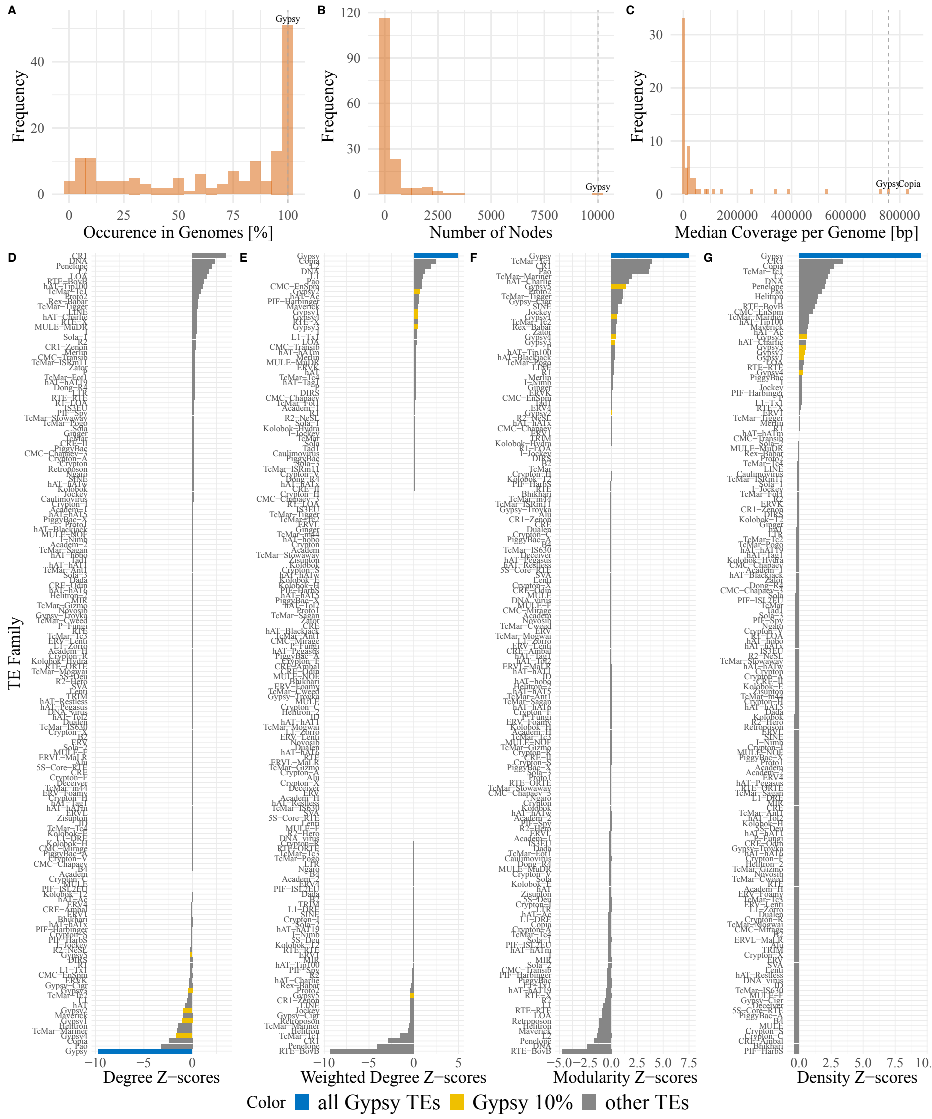

Figure S9: The influence of Gypsy TEs on network stability.

A Histogram showing the distribution of the number of genomes TE subfamilies are found in (maximum is 143). Gypsy is highlighted (143).
B Histogram showing the distribution of the number of nodes (equals number of sequences in the custom library) TE subfamilies have in the bipartite network. Gypsy is highlighted.
C Distribution of the median length [bp] of TE subfamilies with Copia and Gypsy highlighted.
D Bar diagram showing the Z-scores for network degree for each network with one TE subfamily removed. Networks that are created without any Gypsy subfamilies (blue) and without 10% of Gypsy TE subfamilies (yellow) are highlighted.
E Bar diagram showing the Z-scores for network weighted degree for each network with one TE subfamily removed. Networks that are created without any Gypsy subfamilies (blue) and without 10% of Gypsy TE subfamilies (yellow) are highlighted.
F Bar diagram showing the Z-scores for network modularity for each network with one TE subfamily removed. Networks that are created without any Gypsy subfamilies (blue) and without 10% of Gypsy TE subfamilies (yellow) are highlighted.
G Bar diagram showing the Z-scores for network density for each network with one TE subfamily removed. Networks that are created without any Gypsy subfamilies (blue) and without 10% of Gypsy TE subfamilies (yellow) are highlighted.

10 Figure S10

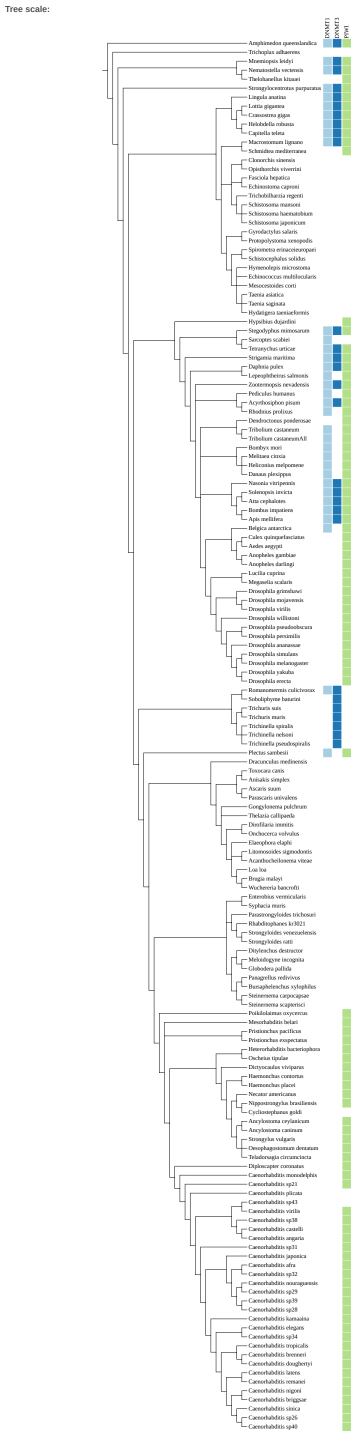

Figure S10: DNMT and PIWI Reciprocal blast search overview.

NCBI guide phylogenetic tree of all the species in the bipartite network. Coloured boxes on the sides indicate the presence of reciprocal blast hits for DNMT1 orthologues (light blue), DNMT3 orthologues (dark blue) and Argonaute Piwi proteins (green).

11 Figure S11

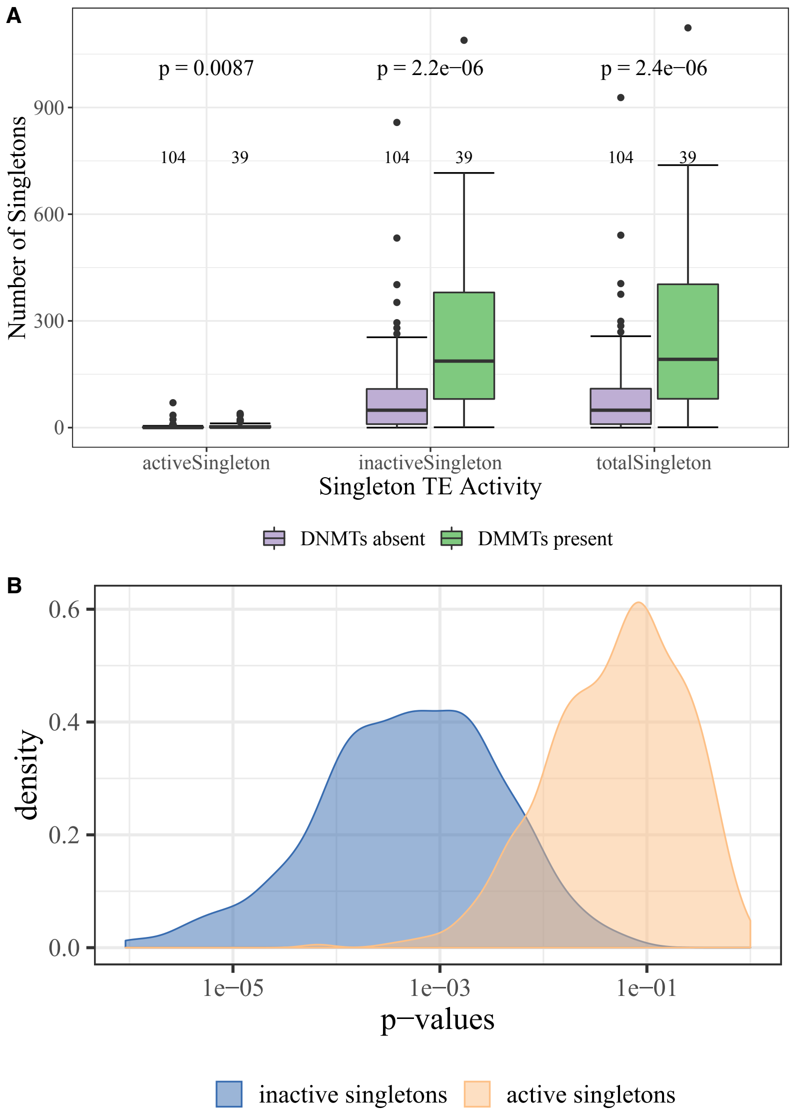

Figure S11: Further investigation of the relationship between singletons and DNMTs.

A Boxplots showing the difference in number of singletons between species with DNMT orthologous and without DNMT orthologues. The numbers on top of the boxes indicate the number n of data points (genomes). P-values are calculated using the Wilcoxon method. The box outlines the IQR with the median shown as a line, whiskers indicate the fences (lower fence: lowest value at most Q1-1.5*IQR, upper fence: largest value no further than Q3+1.5*IQR) and data beyond the whiskers are drawn as individual points (outliers).
B The distributions of the p-values on a log10 scale after bootstrapping the singleton data (p¡0.001, Wilcoxon).

12 Figure S12

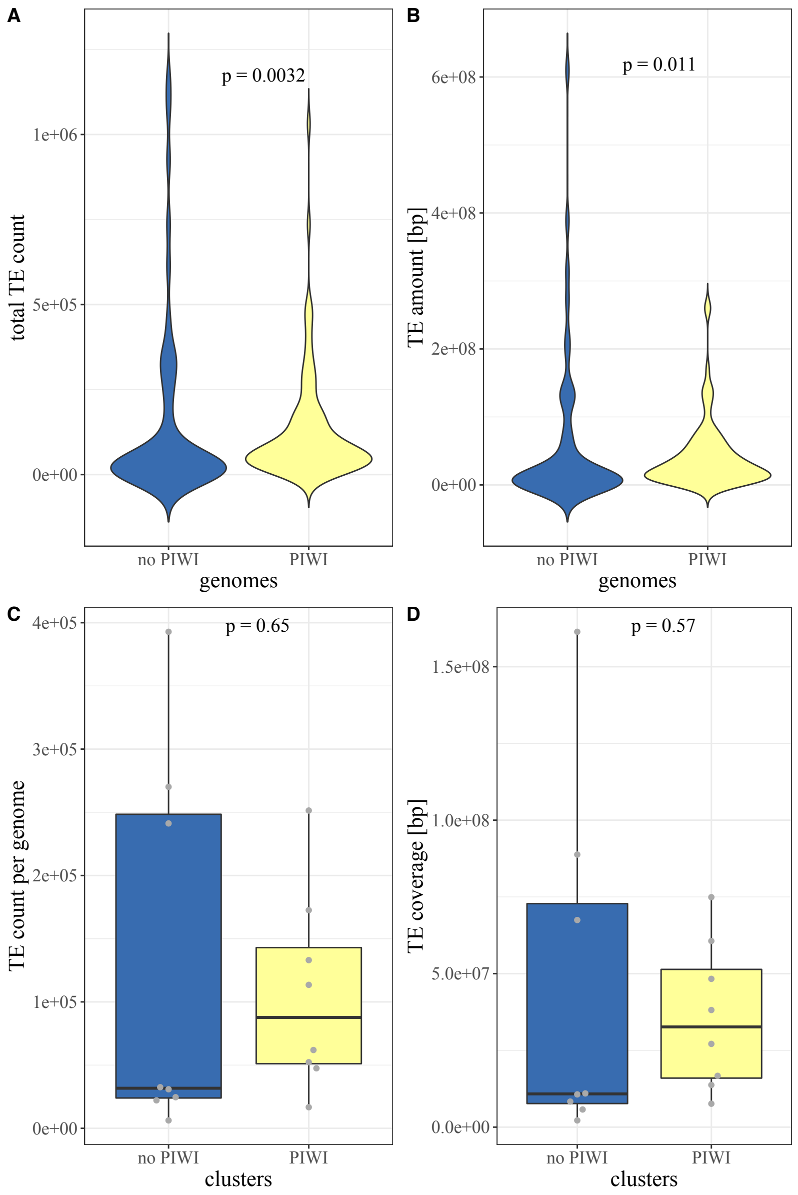

Figure S12: TE data analysis and network analysis.

A Violinplot of the total number of TEs found in species with PIWI (yellow) and without PIWI (blue) (p=0.0032, Wilcoxon). The number of species is displayed above the violin.
B Violinplot of the total sequence length [bp] covered in TEs in species with PIWI (yellow) and without PIWI (blue) (p=0.011, Wilcoxon). The number of species is displayed above the violin.
C Boxplot of the total number of TEs found in species with PIWI (yellow) and without PIWI (blue) averaged by their ortho cluster (p=0.65, Wilcoxon). Each point is one ortho cluster. The box outlines the IQR with the median shown as a line, whiskers indicate the fences (lower fence: lowest value at most Q1-1.5*IQR, upper fence: largest value no further than Q3+1.5*IQR) and data beyond the whiskers are drawn as individual points (outliers).
D Boxplot of the total sequence length [bp] covered in TEs in species with PIWI (yellow) and without PIWI (blue) averaged by their ortho cluster (p=0.57, Wilcoxon). Each point is one ortho cluster. The box outlines the IQR with the median shown as a line, whiskers indicate the fences (lower fence: lowest value at most Q1-1.5*IQR, upper fence: largest value no further than Q3+1.5*IQR) and data beyond the whiskers are drawn as individual points (outliers).
